# Effects of multiple scattering encountered for various small-angle scattering model functions

**DOI:** 10.1107/S1600576718010816

**Published:** 2018-09-20

**Authors:** Grethe Vestergaard Jensen, John George Barker

**Affiliations:** a Chemical and Biomolecular Engineering/NIST Center for Neutron Research, University of Delaware, 100 Bureau Drive, Gaithersburg, Maryland 20899, USA; b NIST Center for Neutron Research, National Institute of Standards and Technology, 100 Bureau Drive, Gaithersburg, Maryland 20899, USA

**Keywords:** small-angle scattering, multiple scattering effects, data modeling

## Abstract

In small-angle scattering theory and data modeling, it is generally assumed that each scattered ray – photon or neutron – is only scattered once on its path through the sample. This assumption greatly simplifies the interpretation of the data and is valid in many cases. However, it breaks down under conditions of high scattering power, increasing with sample concentration, scattering contrast, sample path length and ray wavelength. For samples with a significant scattering power, disregarding multiple scattering effects can lead to erroneous conclusions on the structure of the investigated sample. In this paper, the impact of multiple scattering effects on different types of scattering pattern are determined, and methods for assessing and addressing them are discussed, including the general implementation of multiple scattering effects in structural model fits. The modification of scattering patterns by multiple scattering is determined for the sphere scattering function and the Gaussian function, as well as for different Sabine-type functions, including the Debye–Andersen–Brumberger (DAB) model and the Lorentzian scattering function. The calculations are performed using the semi-analytical convolution method developed by Schelten & Schmatz [*J. Appl. Cryst.* (1980 ▸). **13**, 385–390], facilitated by analytical expressions for intermediate functions, and checked with Monte Carlo simulations. The results show how a difference in the shape of the scattering function plotted *versus* momentum transfer *q* results in different multiple scattering effects at low *q*, where information on the particle mass and radius of gyration is contained.

## Abstract 2

The means by which multiple scattering contributions in experimental small-angle scattering data can be estimated, and how they can be included in the data analysis, are reviewed and discussed. The multiple scattering effects for a range of relevant model scattering functions are calculated using semi-analytically derived solutions to Hankel transforms as well as Monte Carlo simulations.

## 1. Introduction   

**Figure 1:**
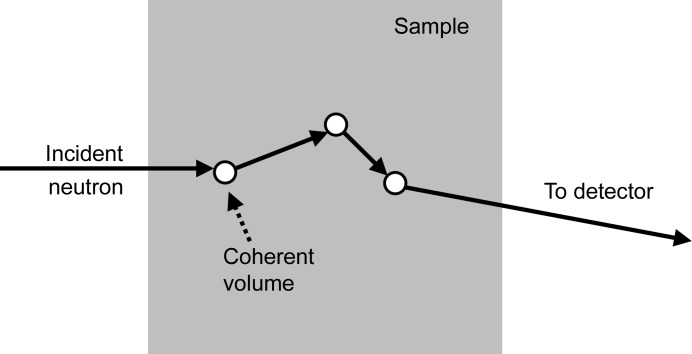
A schematic sketch illustrating a multiple scattering process. An incident ray is scattered by diffraction from within a coherent volume of sample. Subsequently, a widely separated scattering event occurs, with the observed scattering angle depending upon the incoherent addition of the separate scattering events.

Multiple scattering occurs in all small-angle scattering (SAS) experiments to some degree. A schematic illustration of the process is presented in Fig. 1 ▸, showing how multiple scattering from coherent scatterers will give an incoherent contribution to the scattering pattern (Schelten & Schmatz, 1980 ▸). For most typical samples and experimental conditions, the probability of a scattering event is relatively low, and the effect of multiple scattering on the data is therefore negligible. For strongly scattering samples, however, multiple scattering will contribute significantly and must be accounted for to interpret the data correctly. This leads to a less straightforward data analysis and standardized data analysis tools cannot be applied. The multiple scattering effects increase with sample concentration, scattering contrast, size of scattering objects, sample thickness and ray wavelength. Therefore, they are encountered more frequently in small-angle neutron scattering (SANS) than in small-angle X-ray scattering (SAXS) because of the typically larger sample thicknesses (up to 5 mm, compared with 1 mm or less for X-rays) and longer wavelengths (up to 10–20 Å, compared with *ca* 1 Å for X-rays) applied. However, the effects might also be seen in SAXS for particles of high atomic number elements, resulting in a high scattering contrast.

Traditionally, a solution to the multiple scattering problem has been to reduce the sample concentration, thickness or contrast. However, this is not always possible and might also interfere with the structures of interest. The focus of the current work is to clarify how the degree of multiple scattering in experimental data is estimated from sample scattering or transmission (§2.1) and at what level it must be considered in the data interpretation (§2.2), and then to go through the various methods available for the calculation of multiple scattering effects on scattering functions used in data modeling (§3), aimed at enabling full use of the affected data. General results are presented for the case of scattering from spheres (§4.1), together with experimental data illustrating the potential difficulties in determining the correct level of multiple scattering based on transmission measurements (§4.2). Results are also presented for different representative model scattering functions (§4.3), for high-*q* data (§4.4) and for peak scattering (§4.5).

Multiple scattering effects will not appear similar for all types of scattering pattern, and a range of representative types are therefore addressed to illustrate this point: the sphere (Rayleigh, 1910 ▸), Gaussian (Guinier & Fournet, 1955 ▸), Debye–Andersen–Brumberger (DAB; Debye *et al.*, 1957 ▸), Sabine (Sabine & Bertram, 1999 ▸) and Lorentzian scattering functions, where both the DAB and Lorentzian functions are special cases of Sabine functions. The scattering functions including multiple scattering effects were calculated semi-analytically by Hankel transformations, using the convolution method of Schelten & Schmatz (1980 ▸). Useful analytical expressions for the Hankel transforms were derived for each of the scattering functions, leading to faster and more robust calculations. All results were checked with Monte Carlo simulations. On the basis of the results, both the general and the more specific effects of multiple scattering are discussed, including the effects on scattering peaks.

At low momentum transfers *q* [*q* = 4πsin(θ/2)/λ, where λ is the ray wavelength and θ is the total scattering angle], multiple scattering affects the scattering patterns so that the Guinier approximation cannot be used directly to obtain the radius of gyration of the scattering particles. Instead, an apparent value will be obtained. The forward scattering (scattering intensity at *q* = 0), which is directly connected to the total scattering cross section of the scattering particles, will also be modified by multiple scattering, which could lead to erroneous conclusions on the particle mass. Correcting expressions were therefore developed for representative scattering functions to allow determination of the actual radius of gyration, and of the unmodified forward scattering, from experimentally determined values. Simple approximation methods were applied that are valid at the more moderate levels of multiple scattering generally found.

Note that multiple scattering corrections to data regarding the small-angle scattering approximation have already been studied extensively. More complicated general treatments including larger scattering angles have been developed by Vineyard (1954 ▸) and Sears (1975 ▸). Berk & Hardman-Rhyne (1985 ▸) included refraction effects and calculations that extend to very strongly multiply scattering systems. Šaroun (2000 ▸) adapted the Hankel transform method for slit-smeared data obtained from double-crystal diffractometers. Schnablegger & Glatter (1995 ▸) addressed corrections for moderate multiple scattering effects in static light-scattering data. Multiple scattering also has an impact on incoherently scattering samples, such as water or vanadium, which is relevant for their use as standards for the calibration of absolute intensity. This was addressed by Barker & Mildner (2015 ▸), who showed how multiple scattering enhances the scattering at *q* = 0 where the path length through the sample is shortest. Numerous other data treatments have been produced as parts of experimental papers. Herein, we primarily reference treatments that are appropriate for our more limited scope of small-angle scattering following the given scattering model functions.

## 2. Estimating the impact of multiple scattering   

The impact of multiple scattering depends on the scattering power, τ, which, under the assumption of small scattering angles, gives the average number of times any ray – neutron or photon – is scattered on its path through the sample (Ruland & Tompa, 1972 ▸; Berk & Hardman-Rhyne, 1985 ▸). It is therefore a crucial parameter to assess when considering whether multiple scattering effects need to be accounted for in a specific case.

### 2.1. Determining τ   

If the scattering angle is small, the total path length within the sample is constant and equal to the sample thickness *d*_s_. τ is then defined as
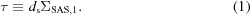
Σ_SAS,1_ is the scattering cross section per sample volume, which represents the probability per unit path length through the sample that a ray is scattered. Σ_SAS,1_ is given by the integral of the scattered function *I*_1_(**q**) over all scattering momentum transfer vectors **q**. *I*_1_(**q**) is here defined as the differential scattering cross section, dΣ_SAS,1_/dΩ, in units of cm^−1^ sr^−1^, where the subscript ‘1’ denotes single scattering, *i.e.* a scattering pattern with no multiple scattering effects. For an isotropic scattering pattern, the integral reduces to an integral over the momentum transfer *q*:
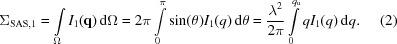

*q*_u_ = 4π/λ is the maximum value of *q*, corresponding to a scattering angle of 180°. Note that the scattering power τ is then proportional to *d*_s_λ^2^. As an alternative to performing the integrals in equation (2), the scattering power τ can also be determined from the small-angle scattering transmission of the sample, *T*_SAS_, which is given by
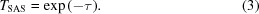



The total sample transmission *T* also contains contributions from absorption, incoherent scattering and inelastic scattering, so that *T* = *T*_abs_ *T*_inc_ *T*_inel_ *T*_SAS_. *T* is determined experimentally from the intensity of the direct beam on the detector, most commonly attenuating the beam to avoid detector damage. Since this measurement will inevitably cover nonzero momentum transfers up to a value *q*_L_, it might also cover a significant fraction of the scattering. In that case, the measured transmission *T*_meas_ will only partially include the contribution *T*_inc_ *T*_inel_ *T*_SAS_, and the included small-angle scattering contribution can be obtained by replacing the lower limit in the integrals in equation (2) by *q*_L_. By making two different transmission measurements, *T*(*q*_L1_) and *T*(*q*_L2_) with suitably different *q*_L_, so that the small-angle scattering mainly contributes in the interval *q*_L1_ < *q* < *q*_L2_, *T*_SAS_ can be estimated from their ratio: *T*_SAS_ ≃ *T*(*q*_L1_)/*T*(*q*_L2_). Many instruments are capable of this and routinely run such transmission measurements. For example, double-crystal instruments often make measurements with and without an analyzer (Schwahn & Yee-Madeira, 1987 ▸). Instruments that use large two-dimensional detectors can determine the two different transmissions *T*(*q*_L1_) and *T*(*q*_L2_) by summing over only the area of the primary unscattered beam and over the entire detector, respectively.

### 2.2. Assessing the multiple scattering impact   

**Figure 2:**
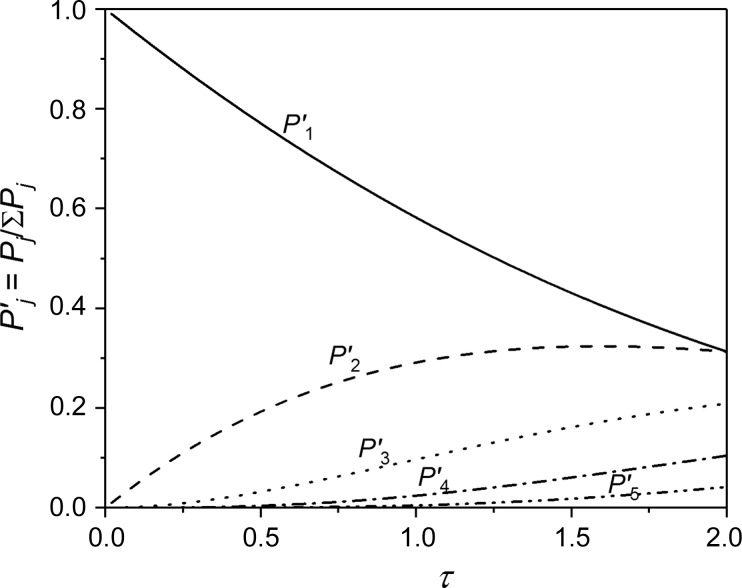
Normalized probability of scattering for the five first scattering orders as a function of scattering power τ. (For τ = 1, *P*_1_′ = 0.582, *P*_2_′ = 0.291 and *P*_3_′ = 0.061.)

τ directly gives the average number of scattering events for a ray, and also the individual probabilities for each of the higher orders of multiple scattering. Let *P_j_* be defined as the probability that an incident ray is scattered *j* times before leaving the sample (Ruland & Tompa, 1972 ▸). Then
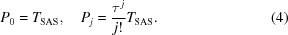
Note that the above expressions are derived assuming that the total path length through the sample is equal to the sample thickness *d*_s_, which is correct for *P*_0_ and *P*_1_, but for higher orders of *P_j_* is only valid for sufficiently small scattering angles, holding reasonably well for SAS data. By renormalizing the above probabilities based only on scattered rays, the normalized distribution over *j* is obtained:
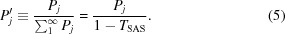
For small τ, double scattering is the dominant contribution to the multiple scattering component, with a normalized probability of *P*_2_′ ≃ *P*_2_/*P*_1_ = τ/2 ≃ (1 − *T*_SAS_)/2. The normalized probabilities for the different scattering orders are shown in Fig. 2 ▸ as a function of τ. Multiple scattering will become significant approximately at a transmission below *T*_SAS_ = 90% corresponding to τ = 0.105, where the normalized probability of double scattering is 5%, *versus* 95% for single scattering. For τ = 1, multiple scattering (*j* ≥ 2) makes up more than 40% of the total scattering.

Scattering powers for samples of spherical particles of different materials (polystyrene, protein, amorphous silica and gold) suspended in water are reported in Table 1 ▸ to illustrate values that might be encountered in typical experiments. They can also serve as a guideline for samples with non-spherical particles of similar size. The values are obtained using the scattering function for spheres as given below in equation (13), giving τ = 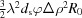, using a sphere radius *R*_0_ = 500 Å and a volume fraction φ = 0.01. Numbers are reported for both neutron and X-ray scattering. For neutron scattering, heavy water (D_2_O) was used as a solvent, which enhances the scattering contrast and limits the contribution from incoherent neutron scattering. A sample thickness of *d*_s_ = 1 mm was applied in all cases. This is a typical value for SAXS, but for SANS, heavy water samples up to 5 mm thick might be used, which would then cause a proportional increase in τ. For SAXS, the contrast increases with the electron density of the particles and the highest scattering power is obtained for gold particles, where multiple scattering would be highly significant. For SANS, the contrast with respect to the deuterated solvent is highest for particles which contain many hydrogen atoms. Here, multiple scattering would be significant for the polystyrene colloid if the sample thickness were doubled to just 2 mm. These are only illustrative examples, and by using larger particles, a higher sample concentration or a larger sample thickness, the scattering power would be significantly enhanced.

For very large particles, one should note that multiple scattering effects might also be accompanied by refraction effects. A ray encounters a phase shift ν upon passing through the particle. If ν is significantly larger than 1, refraction will cause suppression of scattering and a perturbation of the scattering pattern (Berk & Hardman-Rhyne, 1985 ▸). For spheres, the phase shift is given by ν = 2Δρ*R*_0_λ, and refraction effects would set in at *R*_0_ > 4.7 µm for SANS on polystyrene spheres in D_2_O, and at *R*_0_ > 0.8 µm for SAXS on gold spheres in H_2_O.

## 3. Methods for calculation of multiple scattering functions   

Scattering patterns including multiple scattering effects, *I*_m_(*q*), were determined for various different scattering functions *I*_1_(*q*), representing typical scattering patterns that might be encountered. The calculations were performed using the semi-analytical one-dimensional convolution method of Schelten & Schmatz (1980 ▸) as described below. Results were obtained for different scattering power τ and checked by Monte Carlo simulations, which were also applied to perform calculations for scattering functions extending to large scattering angles, where the approximation of scattering at small angles does not apply.

### 3.1. Semi-analytical convolution method   

The multiple scattering function can be determined semi-analytically for a given single scattering function, *I*_1_(*q*). It is given by the sum of the scattering curves for all scattering orders, normalized by their individual scattering cross section Σ_SAS,*j*_ [equation (2] and weighted by their probabilities *P_j_* [equation (4)]. Using the present definition of absolute scale, the total intensity is also normalized by the sample path length and the transmission *T*_SAS_, resulting in the expression
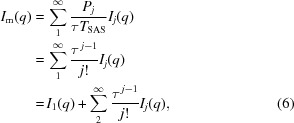
Note that, depending on the method used to determine the sample transmission, the experimental data for *I*_m_(*q*) might be lower by a factor down to *T*_SAS_ if the transmission measurement did not restrict the range of angles collected to sufficiently small momentum transfers *q*_L_.

As shown by Schelten & Schmatz (1980 ▸), any order of two-dimensional scattering function *I_j_*(**q**) is given by the two-dimensional scattering convolution of the next lower order *I*_*j*−1_(**q**) with the first order *I*_1_(**q**):
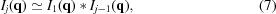
where * symbolizes convolution in two dimensions. This results in a convolution from *I*_1_(**q**) to *I*_m_(**q**) by ‘forward’ and ‘back’ two-dimensional Fourier transforms. Monkenbusch published a program performing both the convolution from *I*_1_(**q**) to *I*_m_(**q**) and the deconvolution back to *I*_1_(**q**) using fast Fourier transform algorithms (Monkenbusch, 1991 ▸). Two data sets were collected for the same sample, but with different sample thicknesses and hence different scattering powers. The single scattering functions obtained by deconvolution were similar, but not identical, illustrating both the power of this approach and also the limitations for real data with noise and a limited *q* region.

Schelten & Schmatz (1980 ▸) gave the expressions for isotropic scattering patterns *I*_1_(*q*) based on a Hankel transform to obtain the intermediate function *i*_1_(*r*), which can then be modified into *i*_m_(*r*) to account for the multiple scattering and transformed back to the multiple scattering function *I*_m_(*q*):
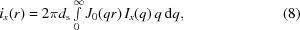


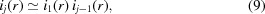


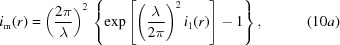


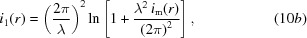


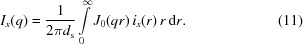

*J*_0_() is the zero-order Bessel function of the first kind and *x* denotes either 1 or m for single or multiple scattering, respectively. Note that, in the limit τ → 0, *i*_m_(*r*) = *i*_1_(*r*) and *I*_m_(*q*) = *I*_1_(*q*), as expected. Equations (8)–(11) [Disp-formula fd10a] [Disp-formula fd10b] [Disp-formula fd11] are modified from the versions of Schelten and Schmatz, where the single scattering functions were given by *S*(*q*), such that *I*_1_(*q*) = [exp(τ)/*d_x_*]*S*(*q*), in accordance with our definition of intensity, which is normalized by the transmission and sample thickness.

Analytical Hankel transform pairs *I*_1_(*q*) 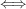 *i*_1_(*r*) exist for all the scattering functions addressed here, such that the forward integral of equation (8) can be solved analytically for *i*_1_(*r*). This greatly improves both the robustness and the speed of the calculations. They are all presented in Appendix *A* [App appa]. For the Gaussian function, the integral of the back transform could also be solved analytically, as given in Appendix *A* [App appa]. For the other functions, it was solved numerically.

The method of Schelten and Schmatz might also, in principle, be used to deconvolute data containing multiple scattering effects, to obtain the corresponding single scattering functions *I*_1_(*q*). This would, however, require very precisely determined scattering intensities with very low noise (Schelten & Schmatz, 1980 ▸), collected over a wide *q* range and with the possibility of extrapolation to obtain intensity values for the lowest and highest *q*. For this reason, the inclusion of multiple scattering in the model function is the preferred approach.

Rather than obtaining *I*_m_(*q*) directly from a transformation of *I*_1_(*q*), it can also be calculated as a combination of *I_j_*(*q*) according to equation (6). Only significantly contributing higher-order scattering functions must be included, which in many cases means that inclusion of the second-order scattering function is sufficient. The orders that must be included can be estimated from equation (5) by setting a certain fraction that must be accounted for; for example, if the target is to include 99% of the scattering, for a given value of τ, it must apply that ∑_*j*=1_ *P*_*j*_′ ≥ 0.99. The scattering power is determined from the transmission *T*_SAS_ [equation (3)] or from the integral over the data or model according to equations (1) and (2). *I_j_*(**q**), and hence *I_j_*(*q*), can be determined numerically from any model function *I*_1_(*q*) by two-dimensional convolutions of *I*_1_(**q**), according to equation (7). They might also be determined by Hankel transforms using equations (8), (9) and (11). In order to determine *I_j_*(*q*) analytically, the transform pairs *I_j_*(*q*) 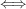 *i_j_*(*r*) = [*i*_1_(*r*)]*^j^* must exist and the back transform given in equation (11) must be solved. Analytical expressions exist for all higher-order transforms *I_j_*(*q*) of the Gaussian function and the Sabine function, and for the second-order Lorentzian transform. All these transform solutions are given in Appendix *A* [App appa]. Alternatively, *I_j_*(*q*) can also be determined by simulation. After combining the contributing orders *I_j_*(*q*) according to equation (6), the result can be compared with the experimental data.

Relatively fast calculation of *I*_m_(*q*) can be achieved either by numerical solution of the Hankel transforms, potentially aided by analytical solutions for *i*_1_(*r*), or by summing contributing orders *I_j_*(*q*), obtained by Hankel transforms or two-dimensional convolutions. This allows for inclusion of the calculation in a model optimization routine, so that the multiple scattering data can be fitted using a structural model, as is routinely done for single scattering data. Apart from the model parameters, this then also requires a parameter giving the scattering power, either determined from transmission measurements or, ideally, obtained from the model using equation (2).

### 3.2. Monte Carlo simulation method   

Simulations can be a very useful tool in the interpretation of experimental data including multiple scattering effects. For any given structural model, simulations give the ability to probe the multiple scattering function *I*_m_(*q*), as well as any individual order of scattering function *I_j_*(*q*). This option is included in the *Igor* software SAS data reduction and analysis macro (Kline, 2006 ▸).^1^ The procedure is time consuming, but running it for the relevant value of scattering power and a series of tentative structural parameters might allow for estimation of the structure that best fits the data.

Monte Carlo simulations were used to verify the results obtained from the faster semi-analytical convolution approach by Schelten and Schmatz in the Lorentzian case, where significant scattering occurs at larger scattering angles so that the small-angle approximation is no longer valid. The simulation approach also allowed for truncation of the function at a scattering angle of 180°, which is not possible using the approach of Schelten and Schmatz. An ideal non-divergent monochromatic beam was applied, scattering from a sample of infinite slab geometry with a thickness of 1 mm, using *ca* 10^7^ scattered rays per simulated scattering pattern. Simulations were also completed to verify calculations for the other scattering functions, but none of the data are included here.

## 4. General effects of multiple scattering   

### 4.1. Scattering from spheres   

**Figure 3:**
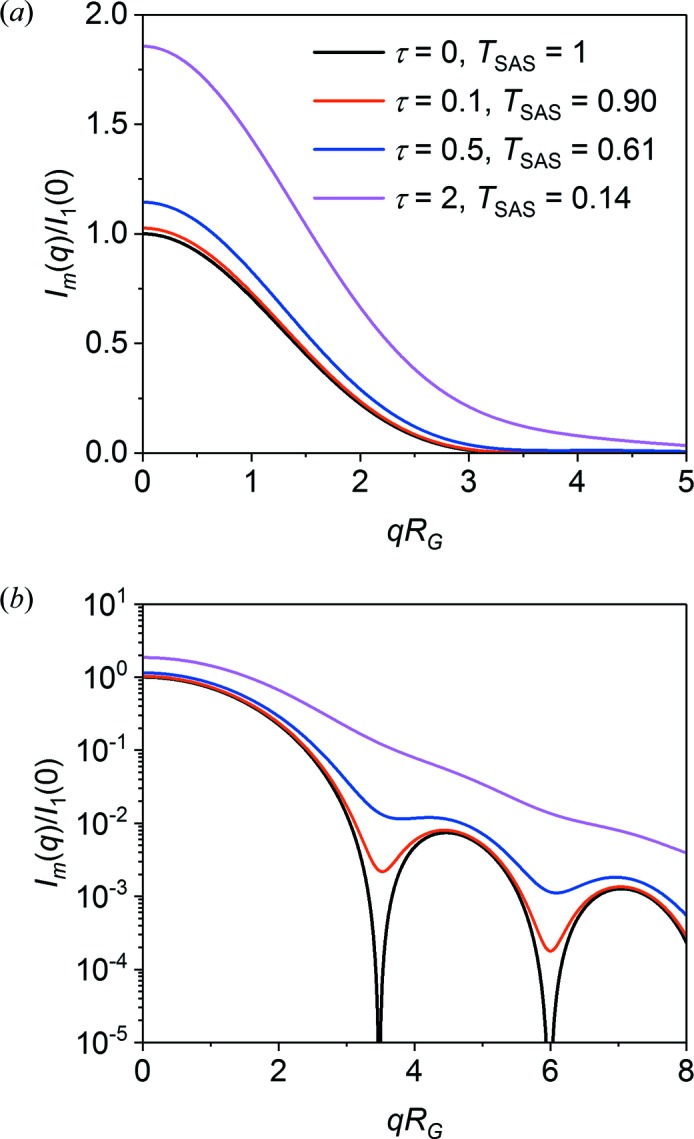
Plots of the multiple scattering function *I*_m_(*q*R_G_) for monodisperse spheres and scattering powers τ = 0, 0.1, 0.5 and 2.0. Panel (*b*) shows *I*_m_(*q*) on a log scale to enhance the minima in the scattering function.

**Figure 4:**
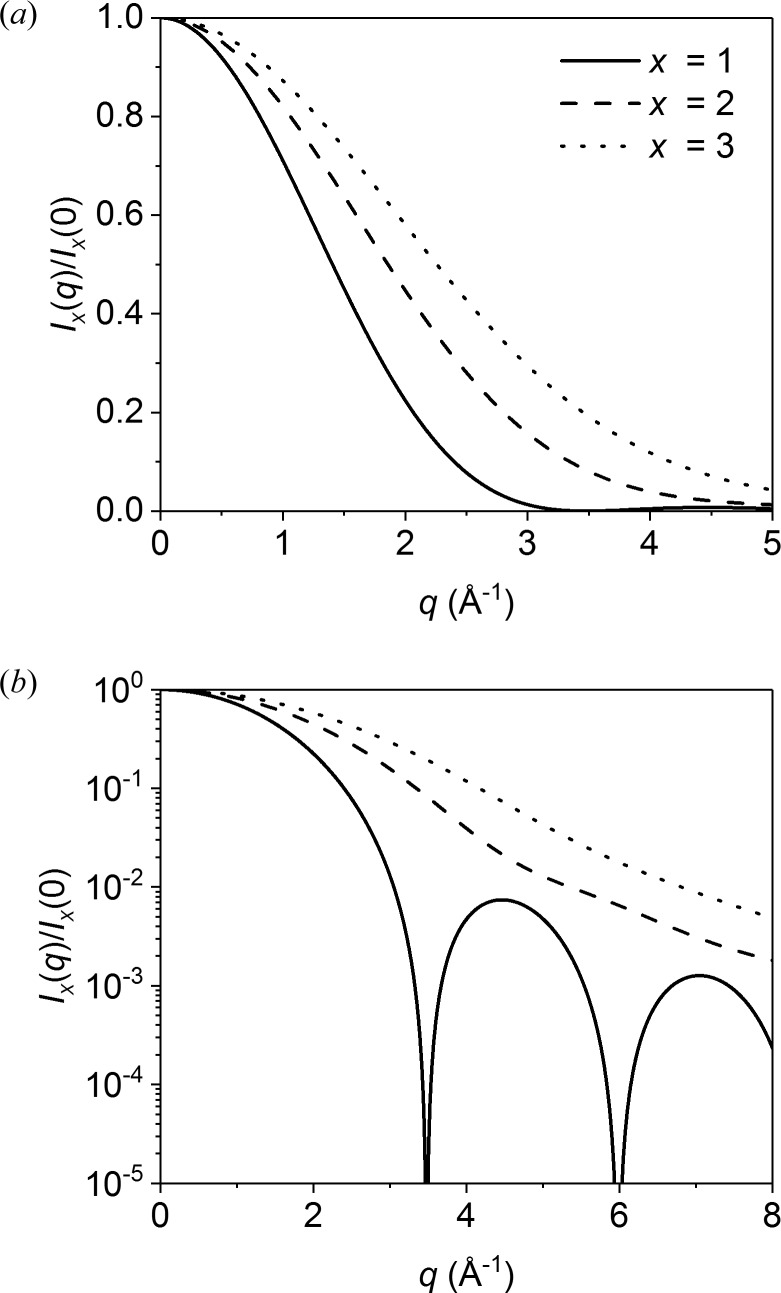
Plots of the single scattering function *I*_1_(*qR*_G_), double scattering function *I*_2_(*qR*_G_) and triple scattering function *I*_3_(*qR*_G_) for monodisperse spheres, all normalized at *q* = 0. Panel (*b*) shows *I*_*j*_(*q*) on a log scale to enhance the minima in the scattering function.

The general effects of multiple scattering can be illustrated by the example of scattering from spheres, as also shown by Schelten & Schmatz (1980 ▸). Fig. 3 ▸ shows *I*_m_(*q*) for scattering from monodisperse spheres of radius *R*_0_ = 500 Å for several different τ. The single scattering function is given below in equation (13). The *q* scale is renormalized by the radius of gyration *R*_G_, and the plot is shown on both a linear intensity scale, highlighting the Guinier region, and a logarithmic intensity scale, highlighting the low-intensity features at high *q*. The contributions from single scattering, *I*_1_(*q*), and double scattering, *I*_2_(*q*) (both normalized to unity at *q* = 0), as determined from Hankel transforms, are shown in Fig. 4 ▸.

Three main effects of increasing multiple scattering (decreasing scattering transmission) are seen. Firstly, the forward scattering *I*_m_(0) is substantially enhanced at a scattering power of τ = 0.693 (corresponding to *T*_SAS_ = 0.5) and increases further for higher τ. This might lead to an overestimation of the contrast or size of the scattering particles if the effects of multiple scattering are not considered in the data analysis. Note that, if the small-angle scattering falls inside the direct beam, the measured transmission does not include the contribution from *T*_SAS_, and the measured scattering patterns will follow *T*_SAS_ *I*_m_(*q*), resulting in decreasing *I*_m_(0) with increasing τ as shown by Schelten and Schmatz. The data interpretation therefore depends critically upon how the sample transmission is measured.

**Figure 5:**
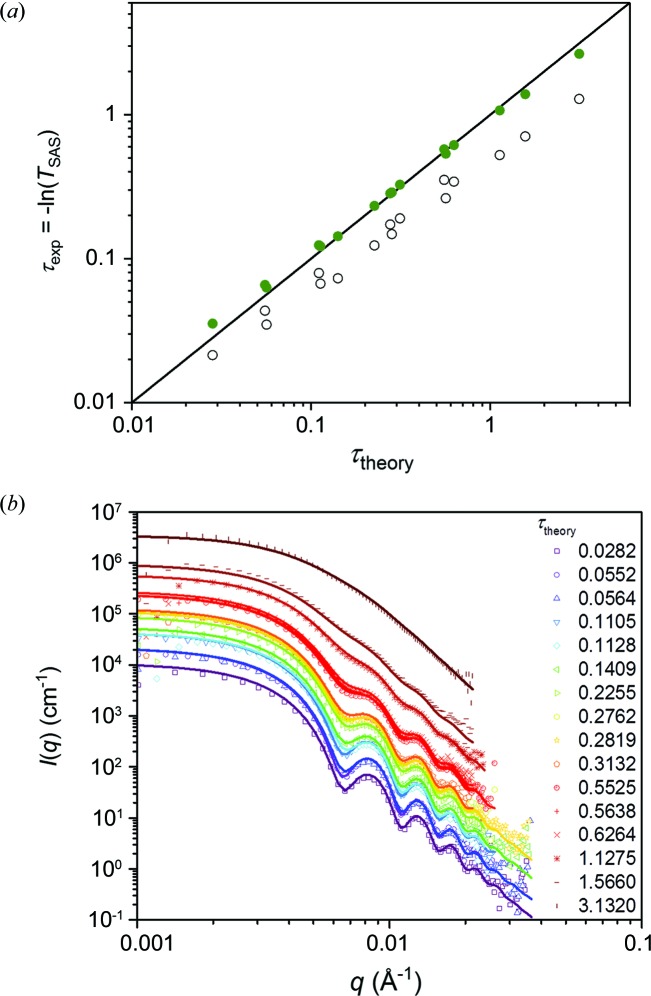
(*a*) Scattering powers for a dilute solution of polystyrene spheres in D_2_O, collected for different values of scattering power in the range 0.028–3.13, obtained by varying the sample thickness and neutron wavelength. The values are determined from transmission measurements (open symbols) and the same values corrected for small-angle scattering in the direct beam (closed symbols), plotted against the theoretical value of the scattering power. The line follows τ_exp_ = τ_theory_. (*b*) Plots of SANS data from the solutions, normalized by the corrected transmissions presented in panel (*a*). The lines are calculated scattering patterns for spheres, including multiple scattering effects according to the theoretical values of the scattering power.

Secondly, the scattering curve is slightly broadened. This is not readily apparent from the plot in Fig. 3 ▸, owing to the moderate degree of multiple scattering, but Fig. 5 ▸ clearly shows that the double scattering curve is much broader than the single scattering curve, with the full width at half-maximum increased by *ca* 30%. At high levels of multiple scattering, this will lead to a noticeable decrease in the apparent radius of gyration. As equation (7) suggests, the apparent radius of gyration for any higher-order scattering function is given by *R*_G,*j*_^2^ = *R*_G_^2^/*j*.

Thirdly, the sharp minima are significantly altered even at a relatively low scattering power, τ = 0.105 (*T* = 0.9), as seen in Fig. 3 ▸(*b*). Fig. 4 ▸(*b*) shows that, already at double scattering, the minima found in the single scattering function are completely washed out. Without considering multiple scattering, this effect might have been assigned to polydispersity or shape anisotropy of the particles.

### 4.2. Assessing multiple scattering effects in experimental data   

When assessing the scattering power for an experimentally determined scattering pattern, a good transmission measurement is key, as described above. However, the high intensities at low *q* resulting from multiple scattering can affect the precision of this measurement, resulting in a wrong estimate of the multiple scattering effect and of the absolute scale of the data, as seen from the data presented here. SANS data were collected on the NGB30 SANS instrument at the National Institute for Standards and Technology Center for Neutron Research (NCNR) (Glinka *et al.*, 1998 ▸) for samples of monodisperse spherical particles of polystyrene (Rennie *et al.*, 2013 ▸) in D_2_O at a volume fraction of φ = 0.0025. The scattering power was varied by varying the neutron wavelength (6, 8.4, 12 and 20 Å) and the sample thickness (1, 2, 5 and 10 mm). A model of monodisperse spheres, including instrumental smearing effects, was fitted to the data set for the lowest scattering power, corresponding to a negligible fraction of multiple scattering of 1 − *P*_1_′ = 0.014. A sphere radius of *R*_0_ = 708.5 ± 0.8 Å was determined, where the uncertainty denotes one standard error. The theoretical values for τ fall in the range 0.028–3.13, as determined from equation (13). Experimental values for τ were calculated from the small-angle scattering transmission *T*_SAS_, which was determined as described above, by the ratio of transmission values measured over the area of the direct beam and over the area of the entire detector. As shown in Fig. 5 ▸(*a*), the determined values did not match the theoretical ones at any considerable scattering power. With increasing scattering power, the small-angle scattering will increase and contribute significantly within the area of the direct beam, leading to a measured transmission which is too high. The ratio between the measured and actual values of *T*_SAS_ is given by
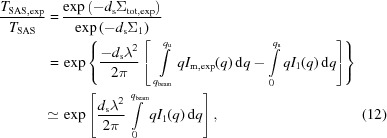
where *q*_beam_ is the largest value of *q* which is considered to fall within the direct beam for the experimental transmission measurement and *q*_u_ is the upper value, as defined under equation (2). The last expression assumes that the scattering cross section for *I*_m_(*q*) has the same relative contribution at *q* < *q*_beam_ as does *I*_1_(*q*). The experimentally determined transmission values were corrected by this factor, using a value for *q*_beam_ that takes into account that they were obtained using a square to define the area of the direct beam. The resulting corrected scattering powers are in much better accordance with the expected values, as also shown in Fig. 5 ▸(*a*). For scattering data in general, it would be necessary to extrapolate an experimentally determined scattering function to *q* = 0 to estimate this correction. This illustrates the difficulty in obtaining precise values of the scattering power from transmission measurements, and hence also the difficulties in obtaining data on an absolute scale. Therefore, we used here the theoretically calculated values for *T*_SAS_ to normalize the data. The data for all scattering powers are shown in Fig. 5 ▸(*b*). The Monte Carlo simulated scattering curves (lines) using the theoretically calculated scattering powers show good agreement with the experimental data, again confirming that the uncorrected scattering powers are not in agreement with the actual conditions.

### 4.3. Multiple scattering effects on different scattering model functions   

Scattering functions modified by multiple scattering *I*_m_(*q*) were calculated as a function of scattering power τ for various representative scattering functions *I*_1_(*q*), listed below. The analytical expressions for the associated scattering power τ are also given, as determined from the integral over *qI*(*q*) as given in equation (2). In addition, approximate solutions τ_approx_ are shown, obtained using an infinite upper limit for *q*, rather than the actual upper value, *q*_u_ = 4π/λ, corresponding to the maximum scattering angle θ = 180°. This modification results in simpler expressions. Owing to the steeply decaying scattering functions *I*(*q*), the deviation from the exact result is negligible, except for the Sabine model with *p* = 1, corresponding to a Lorentzian function.

*Sphere form factor:*

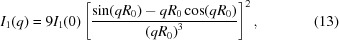


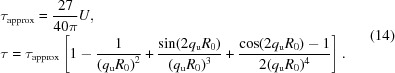



*Gaussian function:*

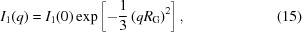


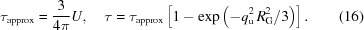



*General Sabine model function (p ≥ 1):*

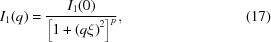


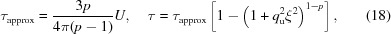
where *U* ≡ 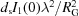 and 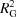 = 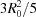 = 3*p*ξ^2^. *R*_0_ is the sphere radius, *R*_G_ is the radius of gyration and ξ is a correlation length. The scattering function from spheres [equation (13)] is the model used most often to describe particulate systems. The Gaussian function [equation (15)] accurately models the Guinier region for any type of particle, but greatly underestimates the scattering at large *q*. The algebraic scattering function, developed by Sabine & Bertram [equation (17)], closely resembles the predicted scattering functions for fractal materials (Sinha *et al.*, 1984 ▸). The DAB scattering function [equation (17), *p* = 2] is commonly used in modeling two-phase materials, and the multiple scattering corrections have also been given by Ruland & Tompa (1972 ▸). The Lorentzian function [equation (17), *p* = 1] has a shape very close to that of the Debye function used to describe the scattering from random-walk statistical polymer chains. The sphere and DAB models both correspond to actual real-space structures, and the forward scattering *I*_1_(0) is directly related to the volume fraction φ of particles, their individual volume *V* and their scattering contrast Δρ, by *I*_1_(0) = φ*V*Δρ^2^. The Sabine model with *p* = 3/2 has the unique property that the shape of all orders of the scattering function is invariant if *q* is rescaled by the beam broadening caused by multiple scattering: *I_j_*(*R*_G,*j*_ *q*)/*j* ^2^ = *I*_1_(*R*_G_ *q*), where *R*_G,*j*_ = *R*_G_/*j* (Sabine & Bertram, 1999 ▸).

**Figure 6:**
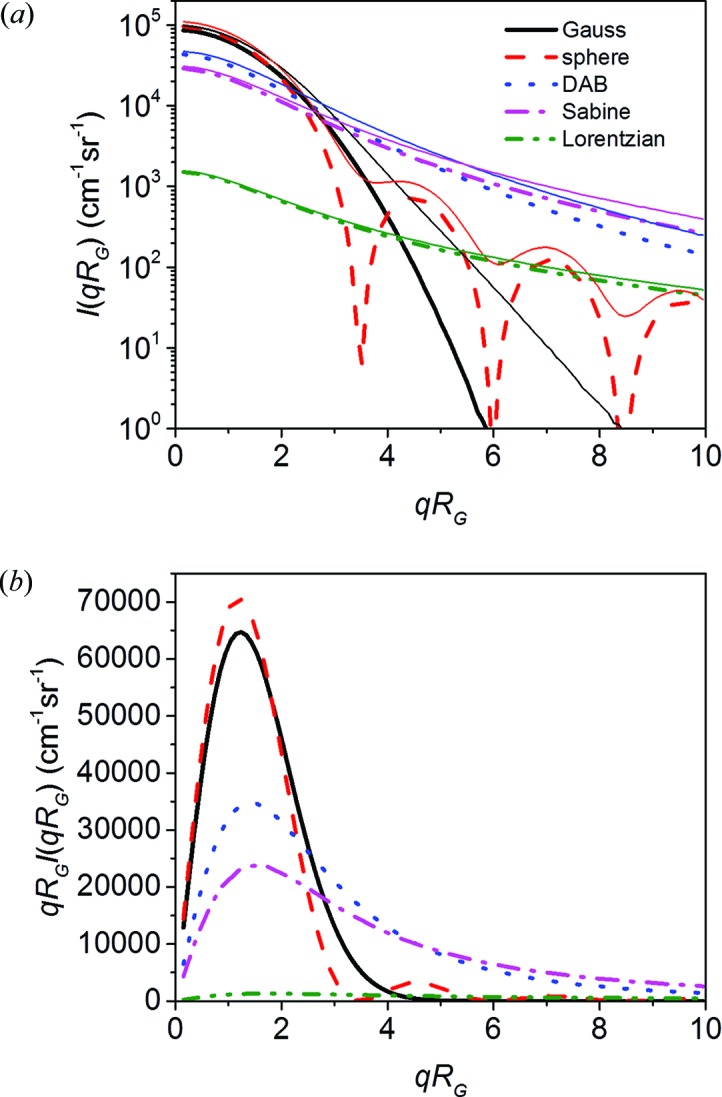
(*a*) Plots of five different scattering functions *I*_1_(*qR*_G_) and of the corresponding multiple scattering functions *I*_m_(*qR*_G_) for τ = 0.5. (*b*) The integrals over *qR*_G_ *I*_1_(*qR*_G_) are proportional to the scattering power τ, according to equations (1) and (2), and therefore all have the same value. For the Lorentzian function an infinite integral will be obtained, and it was therefore truncated at *q*_u_ *R*_G_ = 350 (see text).

The Gaussian function, the sphere function, and Sabine functions for *p* = 2 (DAB model), *p* = 3/2 and *p* = 1 (Lorentzian function) are shown in Fig. 6 ▸(*a*) (thick and dashed lines), together with multiple scattering functions for τ = 0.5 (thin solid lines). The multiple scattering effects are most clearly visible for the sphere function, owing to the smearing of the sharp minima. Fig. 6 ▸(*b*) shows *qR*_G_ *I*(*qR*_G_), which is the integrand in equation (2) for determining the scattering cross section Σ_SAS,1_. The scattering power τ is therefore proportional to the area under the curves. It is seen that the contribution to τ is distributed very differently over *q* for the different scattering functions. For the Lorentzian curve, the integrand does not converge for *q* → ∞, illustrating the significance of the truncation at *q*_u_ = 4π/λ or *qR*_G_ = 4π*R*_G_/λ. The shape of the scattering curve *I*(*qR*_G_) will therefore depend on the value of *R*_G_.

For Fig. 6 ▸ we used *q*_u_ *R*_G_ = 350 for the calculation of τ. Note that the scattering contribution to τ is significant up to *q*_u_, so that only a minor fraction is covered by the plot.

**Figure 7:**
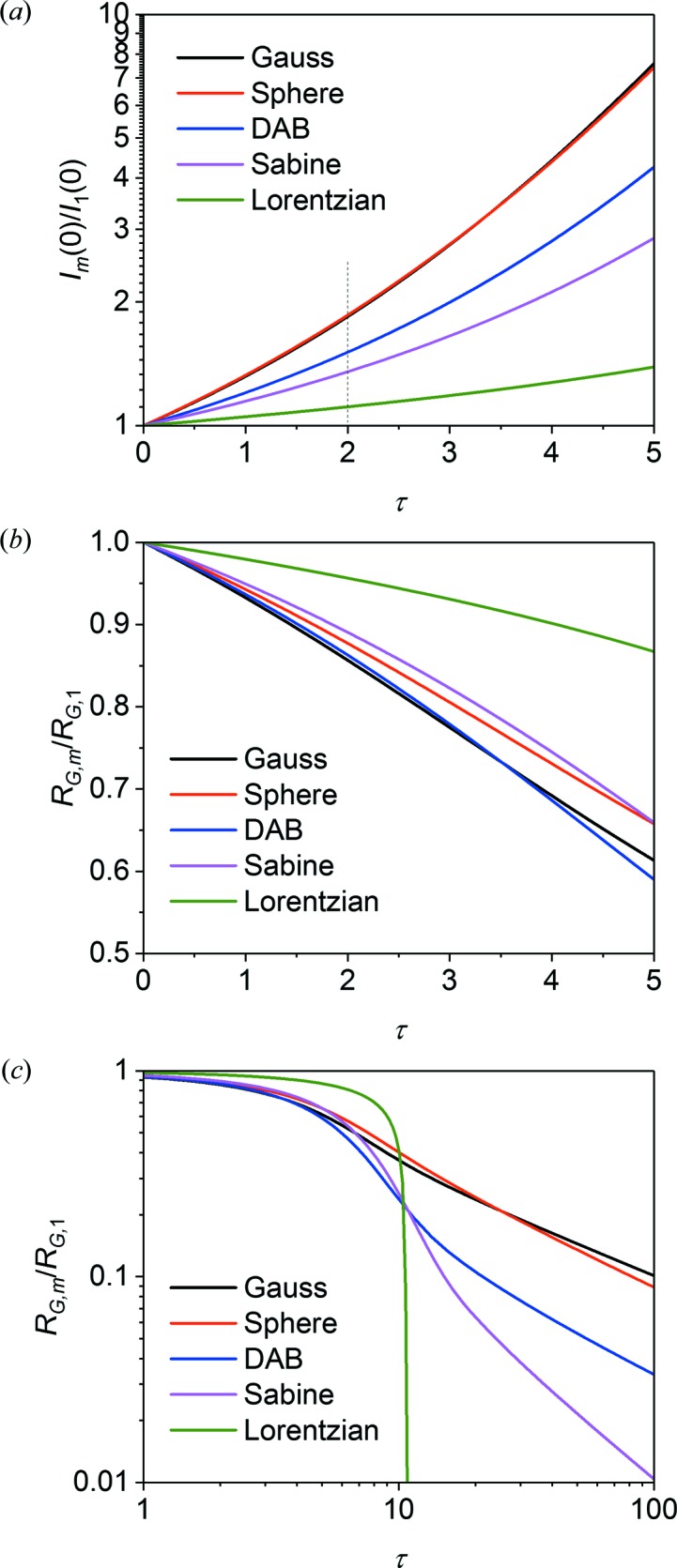
Plots of (*a*) the forward scattering cross section *I*_m_(0) normalized by *I*_1_(0), and (*b*), (*c*) the Guinier radius *R*_G,m_ normalized by *R*_G,1_, *versus* scattering power τ for the five scattering functions. The Lorentzian scattering function was truncated at *q*_u_ *R*_G_ = 350 for calculation of the scattering power τ, which for a wavelength of 6 Å corresponds to a radius of gyration of 167 Å. The power series expressions given in equation (19) fit the results for τ < 2, using the coefficients given in Table 2 ▸.

Multiple scattering functions were determined for the same five functions, using a wide range of values for τ. The forward scattering *I*_m_(0) and apparent radius of gyration *R*_G,m_ are plotted in Fig. 7 ▸. They depend very differently on τ for the five cases, showing that the multiple scattering effects within the Guinier region are duly influenced by the shape of the scattering function at larger *q* beyond the Guinier region. Since the higher-order scattering functions *I_j_*(**q**) are obtained by convoluting *I*_1_(**q**) with itself [equation (7)], it can be expected that the most steeply descending *I*_1_(*q*) will perturb the Guinier region the most. That is, the perturbations are expected to be largest for the sphere and Gaussian functions, smaller for the DAB and Sabine functions, and smallest for the Lorentzian function. This trend is indeed observed for *I*_m_(0). For *R*_G,m_, which reflects the slope of the multiple scattering functions, the trend is less clear, because it depends on the more specific shape of the scattering functions and on the chosen *q* range for which *R*_G,m_ is determined. Analytical solutions for *I*_m_(0) are obtained for the Sabine and Gaussian functions and for *R*_G,m_ for the Gaussian function. They are all given in Appendix *A* [App appa].

The dependence of *I*_m_(0) and *R*_G,m_ on τ as shown in Fig. 7 ▸ was fitted in the range 0 ≤ τ ≤ 2 with empirical power series, similar to the corrections of Boothroyd (1988 ▸) for the second virial coefficient:
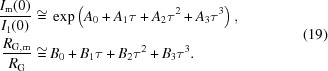
The obtained coefficients are given in Table 2 ▸ for the five different scattering functions, as well as for Sabine functions of intermediate *p*. Then, for a known or estimated τ, and with a rough idea about the type of scattering function, the multiple scattering effect on these quantities can be estimated. In addition, for the given scattering functions it is possible to obtain the forward single scattering *I*_1_(0) and the actual radius of gyration *R*_G_ from the measured values, *I*_m_(0) and *R*_G,m_, using equation (19) together with the fit parameters reported in Table 2 ▸. Copley (1988 ▸) also made similar calculations for spheres using Monte Carlo simulations, which agree well with the current data. The empirical power series break down for τ → ∞. Here, a power law is instead observed for the apparent radius of gyration:
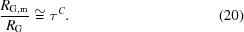
The values of *C* are also given in Table 2 ▸, except for the Lorentzian function which does not follow this behavior.

For the Lorentzian function, the value of τ is determined for a function truncated at *qR*_G_ = 4π*R*_G_/λ. However, the Hankel transformations require continuous functions and are performed for the entire function for *q* → ∞. We therefore applied Monte Carlo simulations to obtain multiple scattering functions for truncated Lorentzian functions using a range of different values of *R*_G_. Only minor deviations from the Hankel transform results were observed in the *q* range covered by Fig. 6 ▸. A larger value of *R*_G_ will give a function which decays less steeply, leading to a weaker impact of multiple scattering for a given scattering power τ. Coefficients for the power law expressions in equation (19) for the truncated Lorentzian functions, as obtained from simulation results, are given in Table 3 ▸, showing the expected trend of decreasing impact with increasing *R*_G_. For real samples scattering according to the Lorentzian function, one might expect the scattered intensity to be effectively truncated at a value of *q* < *q*_u_, given by the length scale of the building blocks of the scattering structure. This would then result in a different effect of multiple scattering compared with the effects reported here, which assume Lorentzian scattering at all *q* up to *q*_u_.

### 4.4. Multiple scattering effects at high *q*   

The effect of multiple scattering at large *q* has been analyzed analytically by Berk & Hardman-Rhyne (1985 ▸) and Monkenbusch (1996 ▸). If the intensity decreases with *q* steeper than *q* ^−2^, it applies that
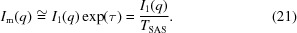
That is, the shape of the scattering pattern is conserved, but the intensity increases with decreasing scattering transmission. For scattering from micrometre-sized structures, multiple scattering is often significant. However, the Guinier region, containing most of the scattering intensity, then typically lies behind the beamstop. Therefore, the transmission *T*_SAS_ is sometimes not accounted for, and the reported scattering pattern will correspond to *T*_SAS_ *I*_m_(*q*), hence closely following the single scattering function *I*_1_(*q*).

### 4.5. Multiple scattering effects on peak scattering   

**Figure 8:**
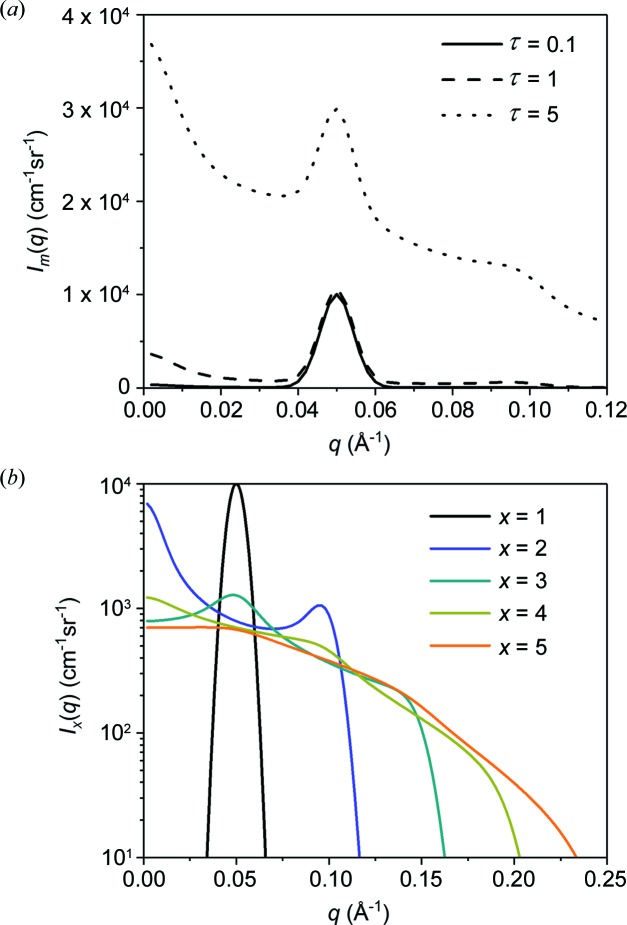
(*a*) Plots of multiple scattering functions *I*_m_(*q*) for a Gaussian-shaped peak for τ values of 0.1, 1 and 5. Strong multiple scattering produces a large background, but the peak shape is only slightly altered. (*b*) Individual contributions *I_x_*(*q*) for orders of scattering *x* in the interval 1 to 5.

In phase-separated systems where a well defined repeated length scale exists, scattering rings are observed at the Bragg angle θ_peak_, leading to a peak in *I*(*q*) at the corresponding scattering vector *q*_peak_. Fig. 8 ▸ shows Monte Carlo simulation results for a scattering function given by a Gaussian peak with *q*_peak_ = 0.05 Å^−1^ and a full width at half-maximum of 0.01 Å^−1^. The scattering curves including multiple scattering effects *I*_m_(*q*) were determined for τ = 0.1, 1 and 5 and are plotted in Fig. 8 ▸(*a*). It is seen that the peak is conserved, so that the higher-order scattering functions *I_j_*(*q*) mainly contribute with a smooth background. Fig. 8 ▸(*b*) shows the individual higher-order scattering contributions *I_j_*(*q*). The second order of scattering will convolute the first-order scattering ring with a ring of the same radius. Therefore, increased intensity is observed at *ca q* = 0 and *q* = 2*q*_peak_. The third-order scattering results in another convolution, and the features will preferentially be ‘scattered back’ to the original angle θ_peak_, so that a peak is again observed at *q* = *q*_peak_, albeit much less pronounced. Thus, for even *j*, features are present at *ca q* = 0 and *q* = 2*q*_peak_, whereas for odd *j* a peak is observed at *q*_peak_. This effect was observed by Silas & Kaler (2003 ▸) in data for a bicontinuous microemulsion, following the Teubner–Strey model, with a peak representing the characteristic correlation length of the sample. They identified a higher-order peak as an effect of multiple scattering, rather than representing an independent structural feature of the sample, and were able to analyze their data accordingly using the method of Schelten and Schmatz.

## 5. Conclusions   

Methods to account for contributions from multiple scattering and the associated error in data analysis are addressed. The determination of the scattering power, and thereby the level of multiple scattering effects, from experimental data is addressed, highlighting the requirement for precise transmission measurements. The scattering functions including multiple scattering effects *I*_m_(*q*) are determined semi-analytically for representative scattering profiles, using the method of Schelten and Schmatz, together with analytical expressions for the intermediate functions, reported in the present paper, facilitating the calculations.

Model-independent structural information, in the form of the forward scattering *I*(0) and radius of gyration *R*_G_, can be determined from scattering data at low *q* in the Guinier region. For data influenced by multiple scattering, apparent values *I*_m_(0) and *R*_G,m_ will be obtained. In general, multiple scattering will lead to an increase in the forward scattering *I*_m_(0) and a decrease in the apparent radius of gyration *R*_G,m_. The present results show how the perturbation of *I*_m_(0) and *R*_G,m_ depends sensitively on the shape of the scattering function at intermediate and large *q* and is therefore different for the different scattering patterns. Approximate expressions for both *I*_m_(0) and *R*_G,m_ as a function of τ are determined for a range of scattering functions, allowing determination of the unperturbed values *I*(0) and *R*_G_ for a given value of τ.

The individual higher-order scattering functions *I_j_*(*q*) can be determined using two-dimensional autoconvolutions of *I*_1_(**q**), or for one-dimensional functions through the Hankel transforms suggested by Schelten and Schmatz. By including the appropriate contributions from the different orders in scattering models, multiple scattering effects can be accounted for in structural model fits, so that even data containing significant multiple scattering contributions can be quantitatively analyzed and interpreted.

## Figures and Tables

**Table 1:** The calculated scattering contrast Δρ and scattering power τ from typical colloidal samples for SAXS and SANS of dilute solutions (φ = 1%) of spherical particles with radius *R*_0_ = 500 Å suspended in water, and having a sample thickness *d*_s_ = 1 mm For X-rays light water is used for solvent, whereas for neutrons heavy water D_2_O is used to enhance the scattering contrast. For X-rays a wavelength of λ = 1.54 Å is used, corresponding to Cu *K*α radiation, whereas for neutrons λ = 6 Å is used. Note that for neutrons some D/H is exchanged, affecting the contrast. All samples have transmissions from X-ray absorption of above 30%, except for the gold sample which is only 0.7%.

Material	Density (g cm^−3^)	Δρ_SAXS_ (Å^−2^)	τ_SAXS_	Δρ_SANS_ (Å^−2^)	τ_SANS_
Polystyrene	1.05	1.8 × 10^−7^	5.6 × 10^−7^	5.0 × 10^−6^	0.067
Protein	1.35	2.5 × 10^−6^	1.1 × 10^−3^	3.2 × 10^−6^	0.027
SiO_2_	2.3	1.03 × 10^−5^	0.019	2.7 × 10^−6^	0.020
Au	19.32	1.15 × 10^−4^	2.4	1.7 × 10^−6^	0.008

**Table 2:** Fit parameters to the power series expressions of equation (19), obtained for various scattering functions, fitted over the range 0 < τ < 2 Numbers are given to the significant digit without affecting the fit quality. All calculations are based on Hankel transforms, giving high-precision results for *I*(0) and *R*_G_, and the zero-order coefficients are therefore fixed at *A*_0_ = 0 and *B*_0_ = 1. The largest deviation of the fit from the calculation is less than 0.03%, observed at τ = 2. The last column gives fit parameters to the power law expressions of equation (20), obtained for various scattering functions, fitted for τ > 100.

Function	10 × *A*_1_	10^2^ × *A*_2_	10^3^ × *A*_3_	10^2^ × *B*_1_	10^3^ × *B*_2_	10^4^ × *B*_3_	*C*
Gaussian	2.5000	2.436	1.68	−6.2485	−4.977	1.79	−0.504
Sphere	2.5791	2.285	1.40	−5.2929	−4.437	0.91	−0.584
Lorentzian† (*p* = 1)	0.4708	0.234	0.20	−1.9610	−0.925	−0.65	
Sabine (*p* = 1.1)	0.4167	0.159	0.09	−1.6924	−0.600	−0.24	−5.000
Sabine (*p* = 1.25)	0.8334	0.547	0.50	−3.2711	−1.989	−1.04	−2.001
Sabine (*p* = 1.5)	1.2500	1.062	1.11	−4.6867	−3.494	−2.07	−1.018
Sabine (*p* = 1.75)	1.4991	1.421	1.31	−5.4187	−4.259	−2.32	−0.730
DAB (*p* = 2)	1.6663	1.619	1.60	−5.8336	−4.756	−1.77	−0.627

† Scattering power obtained using *q*_u_ *R*_G_ = 350.

**Table 3:** Fit parameters to the power series expressions of equation (19), obtained for simulated multiple scattering functions for Lorentzian functions truncated at different values of *q*_u_ *R*_G_, fitted over the range 0 < τ < 2 Numbers in parentheses () represent the one-standard-deviation error on the last digit. All calculations are based on Guinier fits performed in the interval 0.5 < *qR*_G_ < 1.0. *A*_0_ = 0 and *B*_0_ = 1 are expected for an optimal Guinier fit using data for *qR*_G_ → 0.

*q*_u_ *R*_G_	*A*_0_	*A*_1_	*A*_2_	*B*_0_	*B*_1_
50	−0.02254 (1)	0.068 (3)	0.009 (2)	0.887 (3)	−0.050 (3)
100	−0.02152 (1)	0.056 (3)	0.006 (2)	0.893 (2)	−0.044 (2)
200	−0.02245 (1)	0.0535 (8)	0†	0.895 (3)	−0.039 (3)
500	−0.01769 (1)	0.040 (1)	0†	0.894 (3)	−0.031 (3)
1000	−0.02555 (1)	0.0434 (8)	0†	0.888 (3)	−0.020 (3)
2000	−0.02494 (1)	0.0380 (8)	0†	0.890 (3)	−0.020 (3)

† Fixed at zero in the fit.

## Footnotes

1 Disclaimer: the use of certain trade names or commercial products does not imply any endorsement of a particular product, nor does it imply that the named product is necessarily the best product for the stated purpose.

## Appendix A Transforms and analytical solutions

### A1. Hankel transforms to obtain *i*_1_(*r*) analytically from *I*_1_(*q*) for the addressed scattering functions

Gaussian function:

Sphere function:

where *w_r_* ≡

DAB function (*p* = 2):

Sabine function (*p* > 1):

where *K* is the modified Bessel function.

Sabine function (*p* = 3/2):

Lorentzian function (*p* = 1):

A useful identity for determining the apparent radius of gyration for multiple scattering functions is

### A2. Analytical solutions for multiple scattering functions

#### A2.1. Gaussian function

The scattering of any order can be determined from the expression

The scattering at *q* = 0 is

where *f*(τ) = *E_i_*(τ) − γ − ln(τ). *E_i_* is the exponential integral and γ is Euler’s constant. The apparent radius of gyration, *R*_G,m_, is obtained by determining the curvature in the limit of *q*  0:

#### A2.2. Sabine function (*p* = 3/2)

As shown by Sabine, *p* = 3/2 is a special case where the higher-order scattering functions all have the same shape after rescaling. The scattering of any order can be determined from the expression

The scattering at *q* = 0 is

#### A2.3. Lorentzian function

*i*_2_(*r*) ≃ [*K*_0_(*r*)]^2^. As originally shown by Goyal *et al.* (1983 ▸), the Hankel transform pair is used to solve

where *q*_L_ = (*q* ^2^ + 4/*A* ^2^)^1/2^.
